# The single-cell landscape exploring abnormal T cell states and developmental trajectories in heterogeneous non-Hodgkin lymphoma

**DOI:** 10.1016/j.gendis.2025.101812

**Published:** 2025-08-19

**Authors:** Yuqing Wang, Cong Wang, Dezhi Huang, Chanmin Xiao, Qiqi Zhao, Miao Yu, Zheng Wang, Xi Zhang

**Affiliations:** a Medical Center of Hematology, Xinqiao Hospital of Army Medical University, Chongqing Key Laboratory of Hematology and Microenvironment, Chongqing 400037, China; b State Key Laboratory of Trauma and Chemical Poisoning, Chongqing 400037, China; c Jinfeng Laboratory, Chongqing 401329, China; d Bio-Med Informatics Research Center & Clinical Research Center, Xinqiao Hospital of Army Medical University, Chongqing 400037, China

The aberrant differentiation and state transition of the T cell lineage underpin the pathogenesis of non-Hodgkin lymphoma (NHL). Tumor cells originating from B or T cells form the complicated dynamic network with components in the tumor microenvironment (TME).^1^ To orchestrate a tumor-supportive and immunosuppressive environment, tumor cells mainly build contact-dependent communication and paracrine signaling to reshape non-malignant host cells in the TME and remodel the extracellular matrix (ECM).^2^^,^^3^ Angiogenic switching, T cell dysfunction, and ECM crosslinking in the TME make significant contributions to tumor survival, infiltration, and progression. However, the mechanisms driving these processes remain poorly understood.

In this study, by profiling twelve distinct subtypes across four tissue compartments, we present a comprehensive single-cell transcriptomic landscape of T-cell lineages in NHL to elucidate the cellular and molecular mechanisms of lymphomagenesis and immune escape. Our analysis revealed that diverse developmental pathways and cell fates of memory T cells contribute to the complexity of the NHL immune microenvironment. Notably, CD4^+^ memory T cells follow two main trajectories (Fig. 1A). First, *CCR7*^high^ memory-like CD4^+^ T cells (cluster CD4-C1-CCR7) transit into *CTLA4*^+^ Treg-like cells (cluster CD4-C2-CTLA4). Second, *SELL*^high^ memory CD4^+^ T cells (cluster CD4-C3-SELL) give rise to two malignant-like populations, characterized by high *CXCR6* or high *CXCL13* expression (clusters CD4-C4-CXCR6 and CD4-C5-CXCL13). These two clusters both have high malignancy scores and proliferative potential, and are viewed as pro-tumorigenic characteristics within the TME. Similarly, CD8^+^ memory T cells exhibit bifurcating developmental paths (Fig. 1F). One trajectory leads to highly cytotoxic effector CD8^+^ T cells, while the other culminates in exhausted CD8^+^ T cells. These divergent fates underscore how T cell differentiation shapes the immunosuppressive TME in NHL.

**Figure 1:**
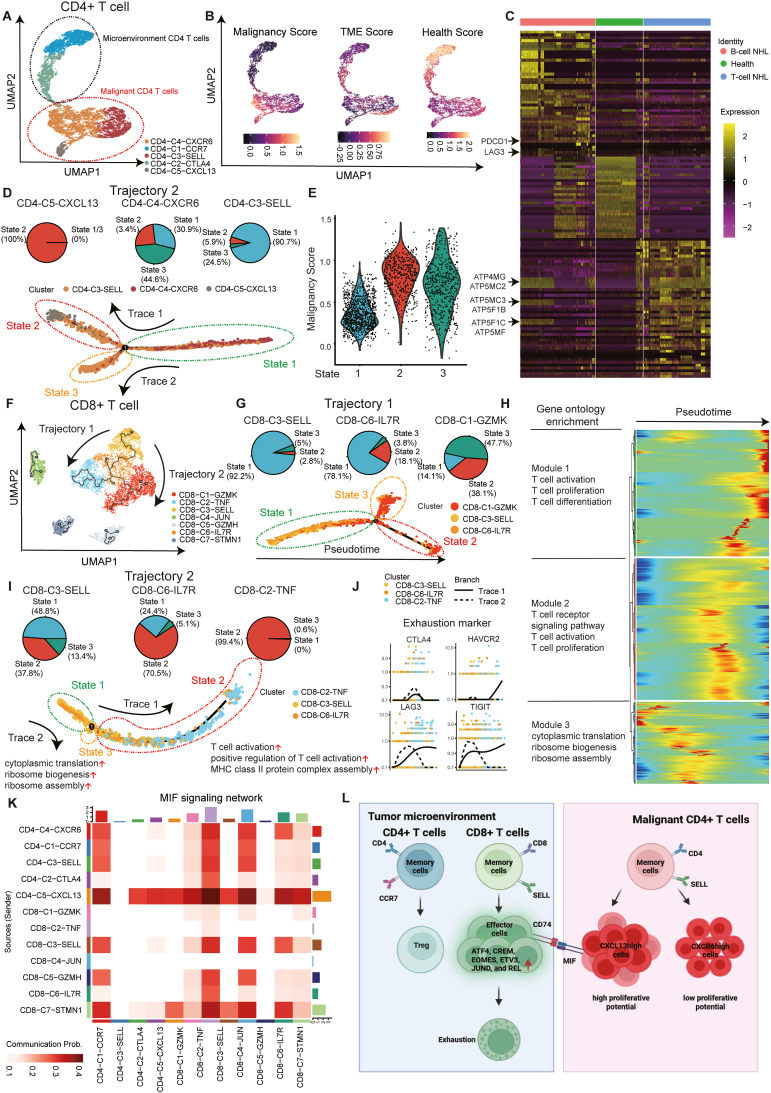
The single-cell landscape exploring abnormal T cell states and developmental trajectories in heterogeneous non-Hodgkin lymphoma. **(A)** UMAP plot visualizing clustering of CD4^+^ T cells colored by cell type. **(B)** UMAP plot visualizing clustering of CD4^+^ T cells colored by malignancy score, TME score, and health score. TME, tumor microenvironment. **(C)** Heatmap visualizing the top markers expressed by each lymphoma type of CD4^+^ T cells. **(D)** Pseudotime trajectory of CD4-C3-SELL, CD4-C4-CXCR6, and CD4-C5-CXCL13 using Monocle 2. **(E)** Violin plot visualizing the malignancy score of three states in the pseudotime trajectory of CD4-C3-SELL, CD4-C4-CXCR6, and CD4-C5-CXCL13. **(F)** Pseudotime trajectories of CD8^+^ T cells using Monocle 3. **(G)** Pseudotime trajectories of CD8-C1-GZMK, CD8-C3-SELL, and CD8-C6-IL7R using Monocle 2. **(H)** Heatmap showing the expression of genes used for pseudotime analysis along the pseudotime trajectory of CD8-C1-GZMK, CD8-C3-SELL, and CD8-C6-IL7R. **(I)** Pseudotime trajectories of CD8-C2-TNF, CD8-C3-SELL, and CD8-C6-IL7R using Monocle 2. **(J)** Dot plots showing exhaustion marker genes according to pseudotime order along the trajectory of CD8-C2-TNF, CD8-C3-SELL, and CD8-C6-IL7R, overlapped with superimposed cell type colors. **(K)** Heatmap showing the relative importance of each cell group of the MIF signaling pathway. **(L)** The landscape of developmental trajectories and the mechanism of CD8^+^ T cell exhaustion in non-Hodgkin lymphoma.

Specifically, CD4^+^ T cells in the NHL milieu segregated into functional subsets (Fig. 1B and C). Within the tumor microenvironment, the *CCR7*^high^ memory-like CD4 (CD4-C1) population has the propensity to differentiate into regulatory T cells (Tregs). This is evidenced by a pseudotime trajectory showing the up-regulation of Treg-associated genes (*CTLA4* and *FOXP3*) and the concomitant down-regulation of memory markers, which indicates a transition toward a CTLA4^+^ Treg-like state (CD4-C2) (Fig. S3B–S3F). Such differentiation enhances the immunosuppressive potential of the TME by amplifying negative regulatory signals. Separately, two malignant-like CD4^+^ subsets emerged, marked by *CXCR6*^high^ (CD4-C4) or *CXCL13*^high^ (CD4-C5) expression (Fig. 1D and E). Particularly, the *CXCL13*^high^ subset contained a higher fraction of proliferating cells and enriched cell cycle-related pathways (mitotic cell cycle and PLK1 signaling) and DNA repair processes (Table S6). The high proliferation and malignancy indexes illustrated the aggressiveness of the *CXCL13*^high^ subset. In contrast, the *CXCR6*^high^ subset was enriched for oxidative phosphorylation, VEGFA–VEGFR2 signaling, and interferon–response pathways, suggesting enhanced metabolic activity and pro-survival signaling in these malignant CD4^+^ T cells^4^ (Table S7).

In the CD8^+^ T cell compartment, chronic stimulation in the lymphoma microenvironment drives a progressive activation–exhaustion program. One developmental trajectory is characterized by increased cytotoxic function (Fig. 1G). Genes involved in T cell activation and antigen presentation (such as those in Module 1) are up-regulated along the trajectory (Fig. 1H), reflecting the heightened effector activity when the tumor burden increases. However, this hyperactivation is not sustainable. As cells follow this trajectory, they eventually down-regulate classical cytotoxic effector genes (*GZMA*, *GZMB*, *GZMK*, and *NKG7*) and simultaneously up-regulate exhaustion markers. The second trajectory similarly led to an exhausted phenotype (Fig. 1I and J). From State 1 to State 2 along the second trajectory, the expression of exhaustion markers (*CTLA4*, *HAVCR2*, *LAG3*, and *TIGIT*) increased (Fig. 1J). In both cases, the data suggest that sustained activation pushes CD8^+^ T cells into dysfunction. They initially become highly cytotoxic but then transit into an exhausted state marked by diminished effector functions. This overactivation-to-exhaustion sequence likely underlies the observed reduction in effective anti-tumor immunity among CD8^+^ T cells in NHL.

Furthermore, our cell–cell interaction analysis revealed the significant role of malignant CD4^+^ T cells in driving CD8^+^ T cell exhaustion. Notably, the highly malignant *CXCL13*^high^ CD4^+^ subset up-regulated macrophage migration inhibitory factor (MIF) and engaged in the CD74/CXCR4 receptor complex on CD8^+^ T cells (Fig. 1K). This MIF-CD74/CXCR4 signaling in the T cell compartment is atypical but likely causes the aberrant activation of CD8^+^ T cells, thus accelerating their entry into exhaustion. In essence, malignant-like CD4^+^ T cells use MIF to overstimulate anti-tumor CD8^+^ T cells, diminishing their long-term efficacy. These findings align with prior observations that MIF can suppress the cytotoxicity of CD8^+^ T cells in cancer.^5^ Therefore, the MIF-CD74/CXCR4 axis represents a novel pathway by which the tumor hijacks T cell interactions to promote immune escape.

In summary, our single-cell study constructed an atlas of T cell states in NHL, revealing an immunosuppressive microenvironment sculpted by divergent CD4^+^ and CD8^+^ T cell trajectories. We identified six key transcription factors (*ATF4*, *CREM*, *EOMES*, *ETV3*, *JUND*, and *REL*) that are highly associated with the induction of T cell exhaustion in lymphoma, suggesting that they act as potential drivers of this dysfunctional state (Fig. S6). Importantly, malignant-like CD4^+^ T cells exhibit unique ligand–receptor interactions (notably MIF-CD74/CXCR4) with CD8^+^ T cells, pinpointing a novel mechanism of inducing CD8^+^ T cell exhaustion (Fig. 1L). These insights illuminate that molecules that modulate the identified transcriptional regulators or disrupt the MIF-CD74/CXCR4 signaling pathway are possible to be targets for therapy. These targets are expected to alleviate T cell exhaustion, reduce immune evasion, and improve treatment outcomes in NHL.

## CRediT authorship contribution statement

**Yuqing Wang:** Writing – original draft, Visualization, Methodology, Investigation, Formal analysis, Conceptualization. **Cong Wang:** Writing – original draft, Methodology, Investigation, Formal analysis, Conceptualization. **Dezhi Huang:** Writing – review & editing, Writing – original draft, Methodology, Investigation, Formal analysis, Conceptualization. **Chanmin Xiao:** Writing – review & editing, Software. **Qiqi Zhao:** Writing – review & editing, Software. **Miao Yu:** Writing – review & editing, Software. **Zheng Wang:** Writing – review & editing, Supervision, Methodology, Conceptualization. **Xi Zhang:** Writing – review & editing, Validation, Supervision, Methodology, Conceptualization.

## Data availability

The data were available upon request from the corresponding author.

## Funding

This work was supported by the Chongqing Science and Health Joint Medical Research Major Project (China) (No. 2022DBXM003), the 10.13039/501100012166National Key R&D Program of China (No. 2023YFC2508905), the Chongqing Technical Innovation and Application Development Special Project (China) (No. CSTB2023TIAD-STX0008), and the Special Project for Talent Construction in Xinqiao Hospital (China) (No. 2022XKRC001) to XZ; the Youth Talent Development Program from Second Affiliated Hospital, Army Medical University (China) (No. 2022YQB014), the Science and Technology Innovation Key R&D Program of Chongqing, China (No. CSTB2023TIAD-STX0001), the 10.13039/501100012166National Key R&D Program of China (No. 2022YFA1103300) to ZW, and the Second Affiliated Hospital of Army Medical University Young Doctor Project (China) (No. 2022YQB101) to CW.

## Conflict of interests

The authors have declared that no competing interests exist.
