# Supplementary material for: The single-cell landscape exploring abnormal T cell states and developmental trajectories in heterogeneous non-Hodgkin lymphoma
Source: Genes Dis. 2025 Aug 19;13(4):101812. doi: 10.1016/j.gendis.2025.101812 (PMC13015217; doi:10.1016/j.gendis.2025.101812)
Supplement: Multimedia component 1 [file mmc1.docx]

**Detailed materials & methods**

**Data selection**

Our search keywords were “single-cell RNA sequencing”, “scRNA-seq”, “lymphoma”, and “non-Hodgkin lymphoma” in PubMed. The platform used single-cell RNA sequencing (scRNA-seq) should be 10X Genomics. The raw count matrix should be publicly free.

**Single-cell data filtering**

Seurat (v4) was used to process the raw-count gene-cell matrix from each database^1^. The cells expressed fewer than 200 genes and more than 2500 genes or exhibited more than 5% mitochondrial genes. These Seurat objects were integrated via the function “IntegrateData”. The integrated matrix was normalized and scaled. Dimension reduction was performed via principal component analysis (PCA). The top 30 principal components (PCs) were further used for uniform manifold approximation and projection (UMAP). Clusters were identified by the function “FindClusters”.

Cells annotated as “T cells” were selected from the raw count matrix. In accordance with the expression of CD4, CD8A, and CD8B, CD4^+^ T cells and CD8^+^ T cells were selected and re-integrated by Harmony^2^. T cells subclusters were identified by the function “FindClusters”. Dimension reduction was performed using PCA. The top 30 PCs were further used for UMAP. Clusters were identified by the function “FindClusters”.

**Cell type annotation**

The integrated matrix including all types of cells was annotated via the function “SingleR” (v2.0.0) function “HumanPrimaryCellAtlasData” and artificially corrected^3^. T cell subclusters were annotated by the combination of the results from the function “FindAllMarkers” and pivotal markers, including memory cells (*CCR7*, *SELL*, and *IL7R*), exhaustive cells (*CTLA4*, *FOXP3*, *HAVCR2*, *LAG3*, and *PDCD1*), Treg markers (*FOXP3*, *CTLA4*, and *LAG3*), and effector markers (*NKG7*, *PRF1*, and *GZMK*).

**Cell state scoring**

The cell state scores were evaluated via the function “AddModuleScore”. The specific genes used are listed in Table S2-4. The cell cycle scores were calculated via the function “CellCycleScoring”.

**Psesuotime analysis**

Monocle 3 (v1.2.9) and Monocle 2 were used for pseudotime analysis^4^. The cell states and trajectories were visualized via a standard Monocle workflow. 50 dimensions were used to perform the function “preprocess_cds”. Dimension reduction was performed via PCA. The reduction method of the function “Embeddings” was UMAP. Two trajectories of CD4^+^ T cells were ordered by the results of the function “differentialGeneTest” with a p-value < 0.01. Two trajectories of CD8^+^ T cells were ordered according to the results of function “FindAllMarkers” with a p-value < 0.05. The dimensions were reduced by the “DDRtree”. The differential expressed genes along branches were identified via the function “BEAM”. Gene enrichment analysis was performed via the function “enrichGO” from clusterProfiler^5^.

**Transcription factor analysis**

pySCENIC (v0.10.0) was used for transcription factor analysis^6^. The ranking references were “hg38__refseq-r80__10kb_up_and_down_tss.mc9nr.genes_vs_motifs.rankings.feather” and “motifs-v9-nr.hgnc-m0.001-o0.0.tbl”.

**Cell-cell communication analysis**

CellChat (v1.1.3) was used to evaluate cell interactions^7^. CellChatDB.human was used as the reference.

**Statistical analysis**

We conducted all the statistical processes using Python (v3.10.8) and R (v4.2.1). Ggplot2 (v3.4.0) and Cytoscape (v3.9.1) were used to construct the figures.

**References**

1. Butler A, Hoffman P, Smibert P, Papalexi E, Satija R. Integrating single-cell transcriptomic data across different conditions, technologies, and species. *Nat Biotechnol*. 2018;36(5):411-420.

2. Korsunsky I, Millard N, Fan J, et al. Fast, sensitive and accurate integration of single-cell data with Harmony. *Nat Methods*. 2019;16(12):1289-1296.

3. Aran D, Looney AP, Liu L, et al. Reference-based analysis of lung single-cell sequencing reveals a transitional profibrotic macrophage. *Nat Immunol*. 2019;20(2):163-172.

4. Qiu X, Mao Q, Tang Y, et al. Reversed graph embedding resolves complex single-cell trajectories. *Nat Methods*. 2017;14(10):979-982.

5. Yu G, Wang LG, Han Y, He QY. clusterProfiler: an R package for comparing biological themes among gene clusters. *Omics J Integr Biol*. 2012;16(5):284-287.

6. Van de Sande B, Flerin C, Davie K, et al. A scalable SCENIC workflow for single-cell gene regulatory network analysis. *Nat Protoc*. 2020;15(7):2247-2276.

7. Jin S, Guerrero-Juarez CF, Zhang L, et al. Inference and analysis of cell-cell communication using CellChat. *Nat Commun*. 2021;12(1):1088.
