# Supplementary material for: The single-cell landscape exploring abnormal T cell states and developmental trajectories in heterogeneous non-Hodgkin lymphoma
Source: Genes Dis. 2025 Aug 19;13(4):101812. doi: 10.1016/j.gendis.2025.101812 (PMC13015217; doi:10.1016/j.gendis.2025.101812)
Supplement: Multimedia component 2 [file mmc2.docx]

**Figure S1. The single-cell transcriptomic landscape of non-Hodgkin lymphoma.** (A) Schematic analysis workflow of the integration and analysis of single-cell RNA-seq datasets of non-Hodgkin lymphoma. Ten scRNA-seq datasets were selected to identify key molecules involved in the malignant transformation of lymphoma. Pie plot visualizing the proportions of (B) disease types, (C) tissue origins, and (D) malignancies in all included cells. (E) UMAP plot visualizing clustering of non-Hodgkin lymphoma cells colored by cell type. (F) Violin plot visualizing the expression of pivotal marker genes of clustering. CTCL: cutaneous T-cell lymphoma; PCNSL: primary central nervous system lymphoma; PCFCL: primary cutaneous follicle center lymphoma; FL: follicle lymphoma; DLBCL: diffuse large B cell lymphoma; SMZL: splenic marginal zone lymphoma; PBMC: peripheral blood mononuclear cells; HC: healthy; NL: non-lesion; MA: malignant; SP, single positive; DN: double negative.

**Figure S2. Single-cell transcriptomic landscape of CD4^+^ T cells of non-Hodgkin lymphoma.** (A) Heatmap visualizing top100 differential expressed genes by each cell type of CD4^+^ T cells. (B) Violin plot visualizing the expression of pivotal marker genes of CD4^+^ T cell clustering. (C) Bar plot visualizing the proportion of tissue origin in each cluster of CD4^+^ T cells. (D) Bar plot visualizing the proportions of lymphoma types in each cluster of CD4^+^ T cells. (E) Violin plot visualizing the memory score of CD4^+^ T cell clustering. (F) UMAP plot visualizing clustering of CD4^+^ T cells colored according to the predicted cell cycle stages. (G) Bar plot visualizing the proportion of proliferative cells in each cluster of CD4^+^ T cells.

**Figure S3. Single-cell development trajectories of CD4^+^ T cells.** (A) Pseudotime trajectories of CD4^+^ T cells using Monocle 3. Pseudotime trajectories of CD4-C1-CCR7 and CD4-C2-CTLA4 using Monocle 2, displayed based on (B) pseudotime and (C) cell cluster. (D) Violin plot visualizing the healthy score and TME score of three states in the trajectory of CD4-C1-CCR7 and CD4-C2-CTLA4. (E) Dot plots showing marker genes according to pseudotime order along the trajectory of CD4-C1-CCR7 and CD4-C2-CTLA4, overlapped with cell type colors superimposed. (F) Heatmap showing expression of genes used for pseudotime analysis along pseudotime of the trajectory of CD4-C1-CCR7 and CD4-C2-CTLA4. (G) Pseudotime trajectories of CD4-C3-SELL, CD4-C4-CXCR6, and CD4-C5-CXCL13 using Monocle 2, displayed based on pseudotime. (H) Violin plot visualizing the healthy score of three states in the trajectory of CD4-C3-SELL, CD4-C4-CXCR6, and CD4-C5-CXCL13. (I) Dot plots showing marker genes according to pseudotime order along the trajectory of CD4-C3-SELL, CD4-C4-CXCR6, and CD4-C5-CXCL13, overlapped with cell type colors superimposed. (J) Heatmap showing expression of genes used for pseudotime analysis along the trajectory of CD4-C3-SELL, CD4-C4-CXCR6, and CD4-C5-CXCL13.

**Figure S4. Single-cell transcriptomic landscape of CD8^+^ T cells of non-Hodgkin lymphoma.** (A) UMAP plot visualizing clustering of CD8^+^ T cells colored by cell types. (B) Violin plot visualizing the expression of pivotal marker genes of CD8^+^ T cell clustering. (C) Bar plot visualizing the proportion of tissue origin in each cluster of CD8^+^ T cells. (D) Bar plot visualizing the proportion of lymphoma type in each cluster of CD8^+^ T cells. (E) UMAP plot visualizing clustering of CD8^+^ T cells colored by predicted cell cycle stages. (F) Bar plot visualizing the proportion of proliferative cells in each cluster of CD8^+^ T cells. (G) Violin plot visualizing the memory score, cytotoxicity score, and exhaustion score of CD4^+^ T cell clustering.

**Figure S5. Single-cell development trajectories of CD8^+^ T cells.** Pseudotime trajectories of CD8-C1-GZMK, CD8-C3-SELL, and CD8-C6-IL7R using Monocle 2, displayed based on (A) pseudotime, and (B) cell state. (C) Dot plots showing marker genes according to pseudotime order along the trajectory of CD8-C1-GZMK, CD8-C3-SELL, and CD8-C6-IL7R, overlapped with cell type colors superimposed. Pseudotime trajectories of CD8-C2-TNF, CD8-C3-SELL, and CD8-C6-IL7R using Monocle 2, displayed based on (D) pseudotime, and (E) cell state. (F) Heatmap showing expression of genes used for pseudotime analysis along pseudotime of the trajectory of CD8-C2-TNF, CD8-C3-SELL, and CD8-C6-IL7R. (G) Dot plots showing marker genes according to pseudotime order along the trajectory of CD8-C2-TNF, CD8-C3-SELL, and CD8-C6-IL7R overlapped with cell type colors superimposed.

**Figure S6. Predicted regulatory network of T cell exhaustion in CD4^+^ T cells and CD8^+^ T cells in non-Hodgkin lymphoma.** (A) Gene selection flow. (B) Heatmap showing expression of regulons among three cellular states of malignant CD4^+^ T cells. (C) Heatmap showing expression of regulons among three cellular states of the trajectory of CD8-C2-TNF, CD8-C3-SELL, and CD8-C6-IL7R in TME. Regulatory network of T cell exhaustion in (D) CD4^+^ T cells and (E) CD8 T^+^ cells.

**Figure S7. Cell communications between CD4^+^ T cells in TME, malignant-like CD4^+^ T cells, and CD8^+^ T cells in TME.** (A) The dot plot showing the outgoing signaling patterns of secreting cells between CD4^+^ T cells in TME, malignant-like CD4^+^ T cells, and CD8^+^ T cells in TME. (B) Heatmap shows the relative importance of each cell group based on the computed four network centrality measures of MHC-II signaling pathway. (C) The relative contribution of each ligand-receptor pair to the overall communication network of MHC-II signaling pathway. (D) The MIF signaling pathway network. (E) Violin plot visualizing the expression of genes involved MIF signaling pathway of non-Hodgkin lymphoma cell clustering. TME, tumor microenvironment.

**Figure S8. Cell communications between CD4^+^ T cells in TME, malignant-like CD4^+^ T cells, and CD8^+^ T cells in TME.** (A) The dot plot showing the incoming signaling patterns of target cells between CD4^+^ T cells in TME, malignant-like CD4^+^ T cells, and CD8^+^ T cells in TME. (B) Heatmap shows the relative importance of each cell group based on the computed four network centrality measures of TNF signaling pathway. (C) The heatmap shows the relative importance of each cell group of TNF signaling pathway. (D) Heatmap shows the relative importance of each cell group based on the computed four network centrality measures of MHC-I signaling pathway. (E) The relative contribution of each ligand-receptor pair to the overall communication network of MHC-I signaling pathway. TME, tumor microenvironment.
