# Supplementary figures and images for: The single-cell landscape exploring abnormal T cell states and developmental trajectories in heterogeneous non-Hodgkin lymphoma
Source: Genes Dis. 2025 Aug 19;13(4):101812. doi: 10.1016/j.gendis.2025.101812 (PMC13015217; doi:10.1016/j.gendis.2025.101812)

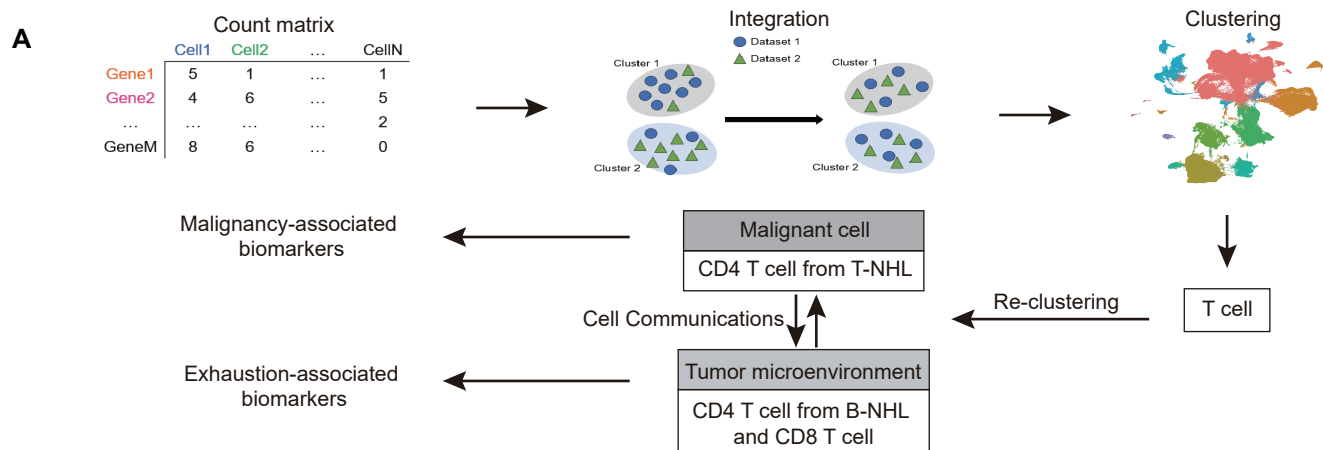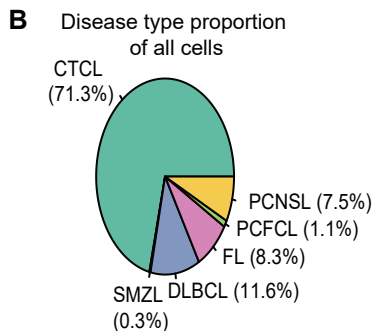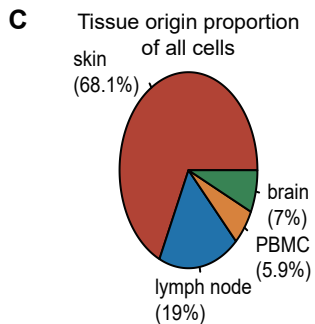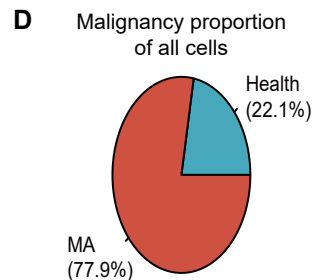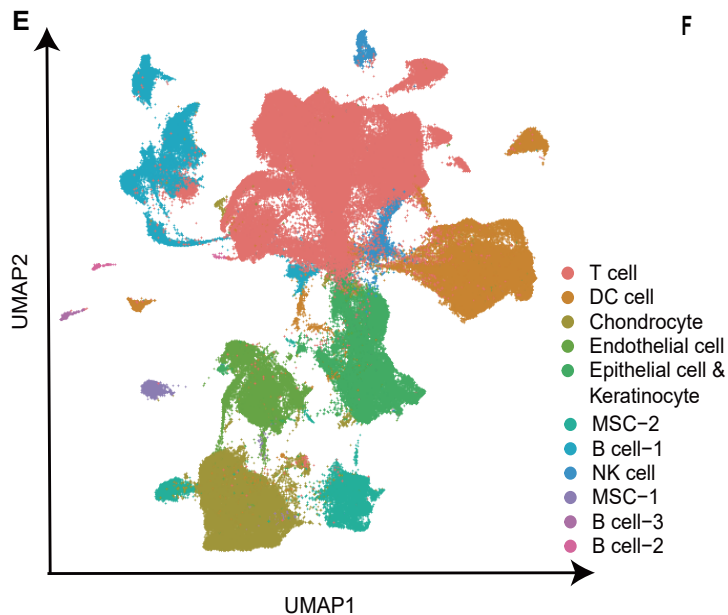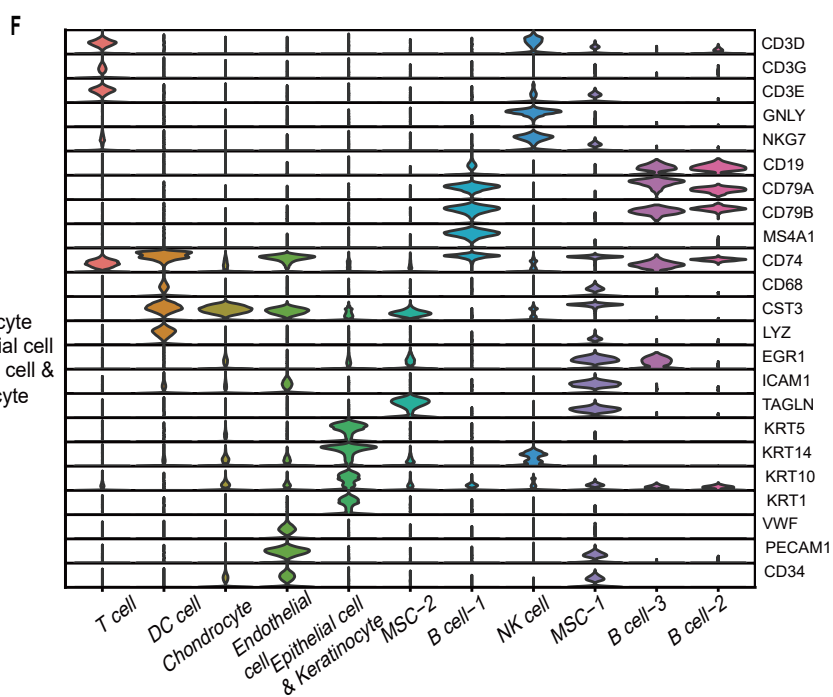

Supplement: Multimedia component 3 [file mmc3.pdf]

A

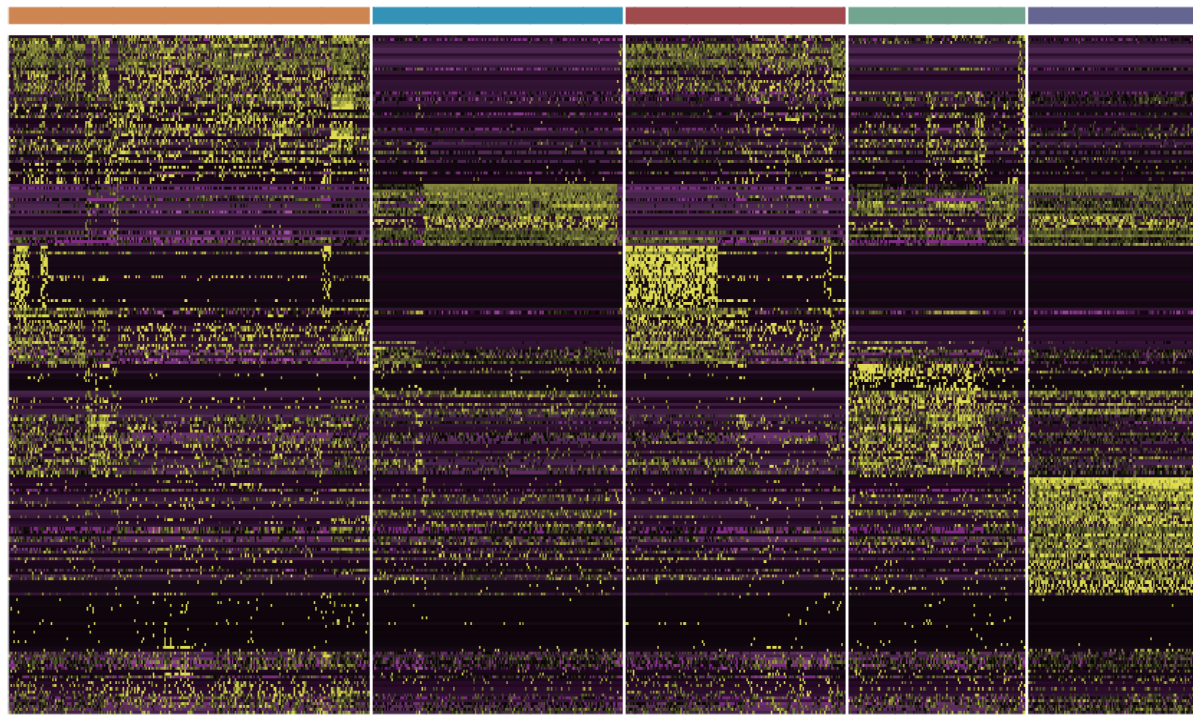

Identity

- CD4-C4-CXCR6
- CD4-C1-CCR7
- CD4-C3-SELL
- CD4-C2-CTLA4
- CD4-C5-CXCL13

Expression

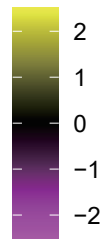

B

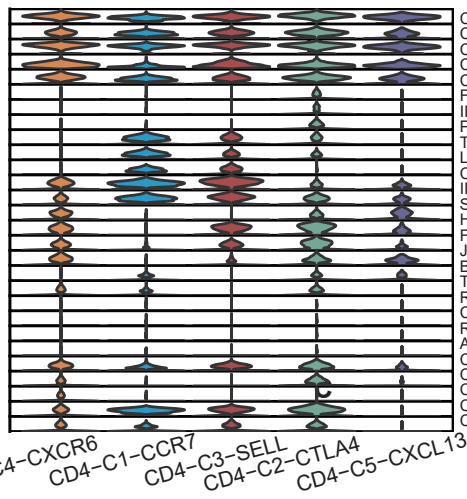

C

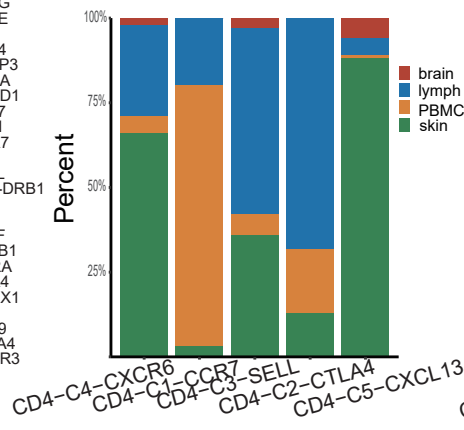

D

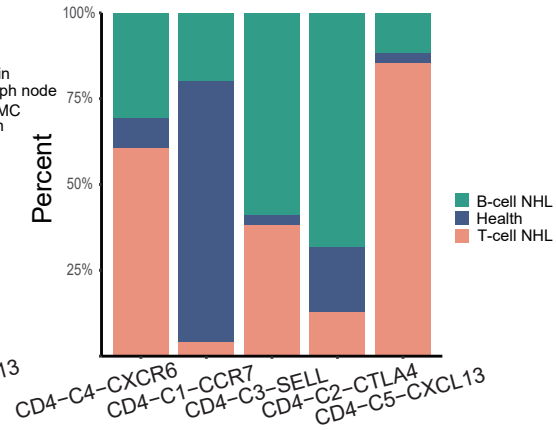

E

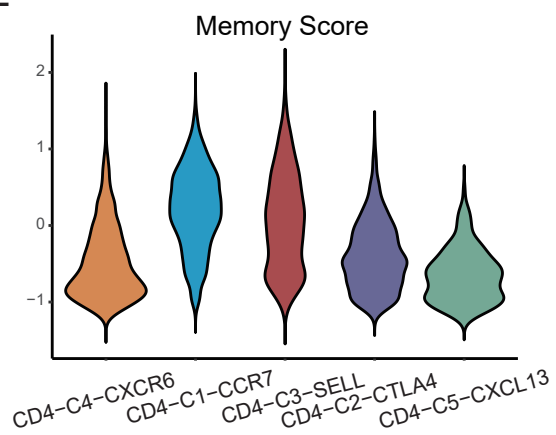

F

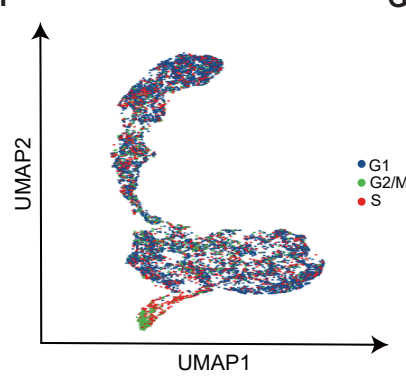

G

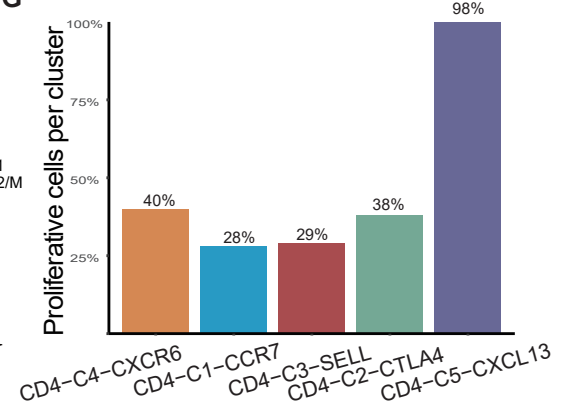

Supplement: Multimedia component 4 [file mmc4.pdf]

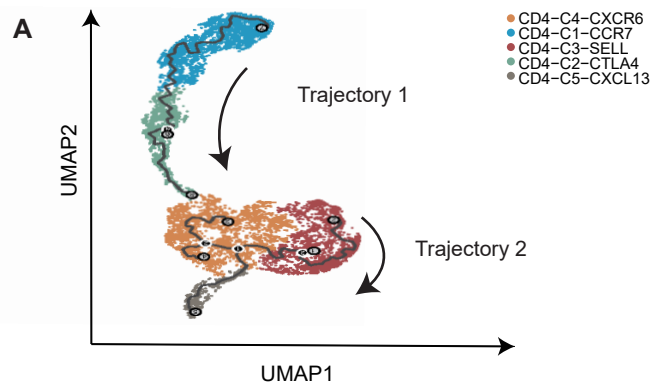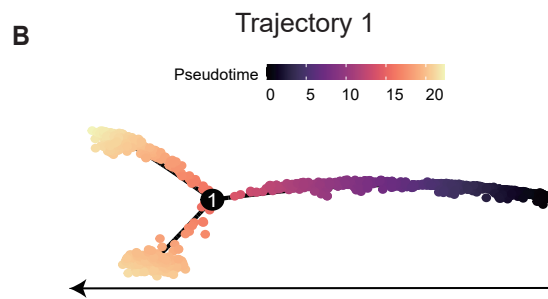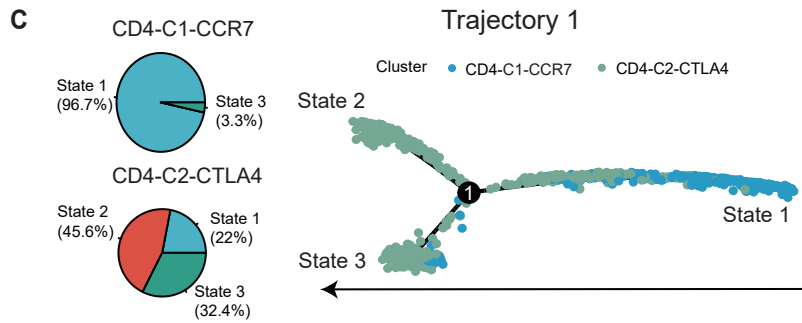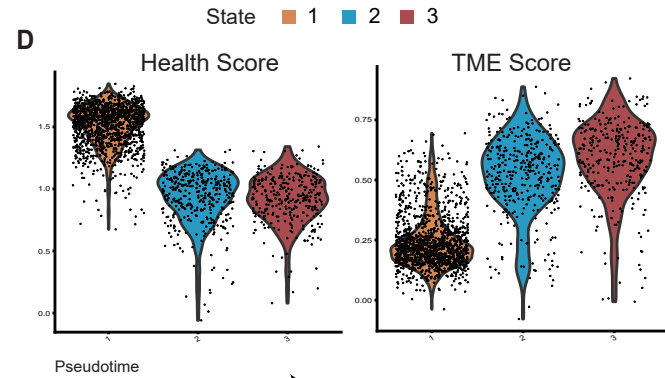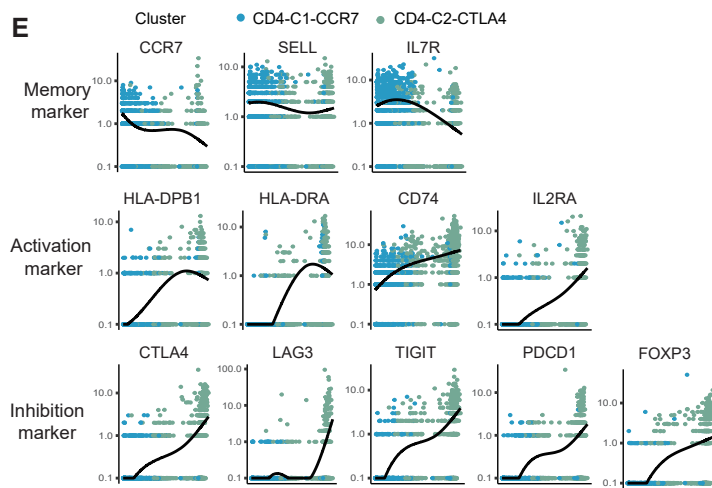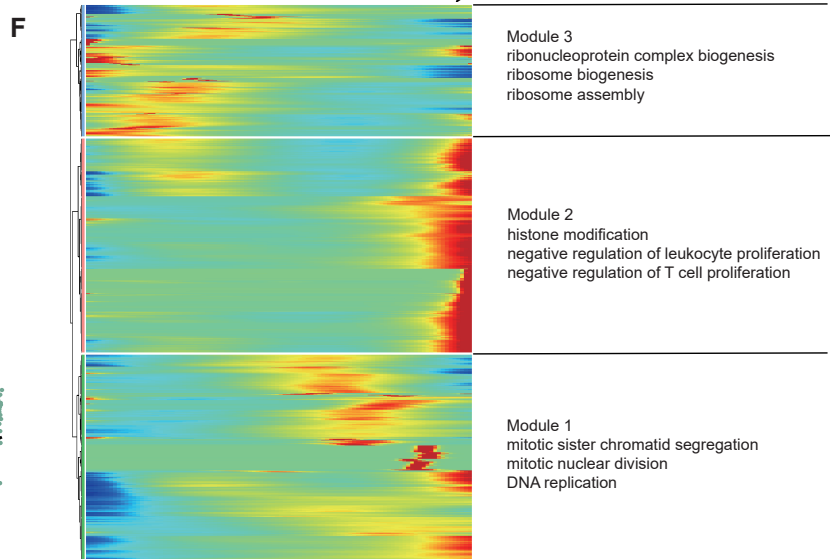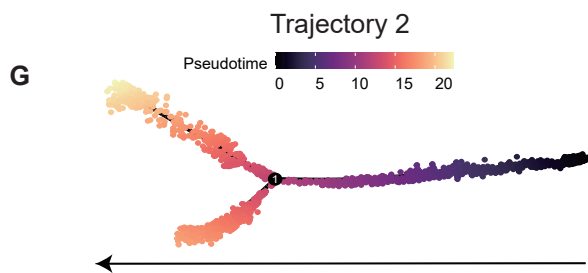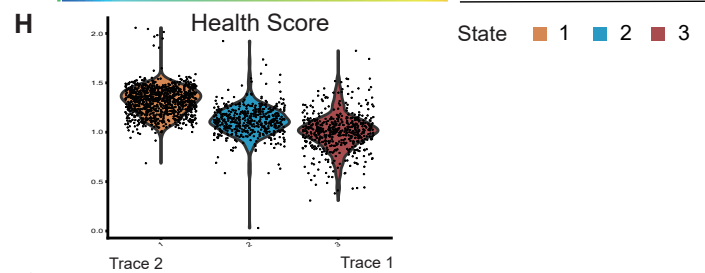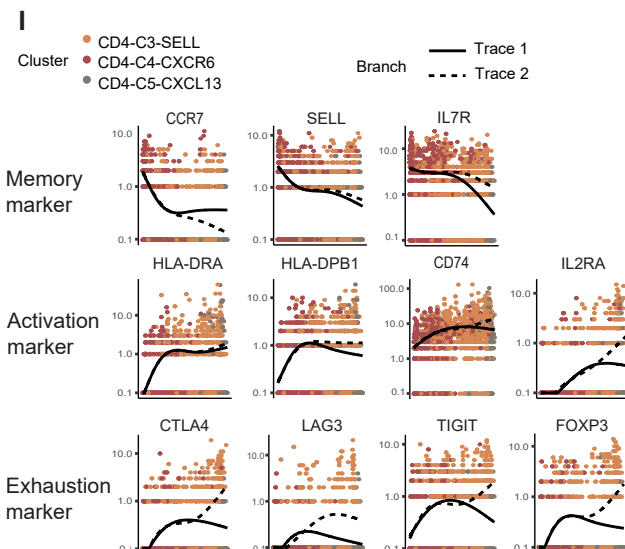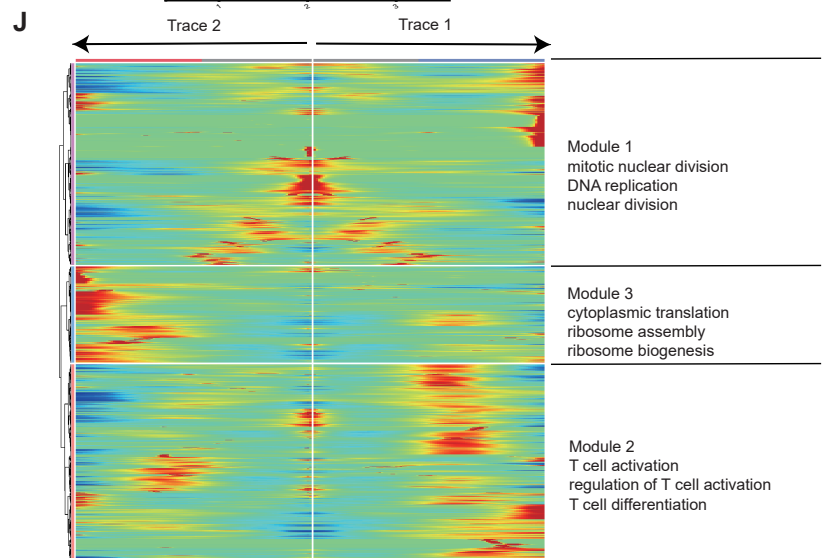

Supplement: Multimedia component 5 [file mmc5.pdf]

A

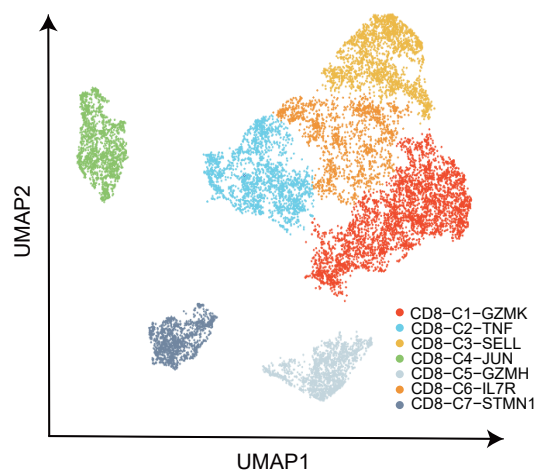

B

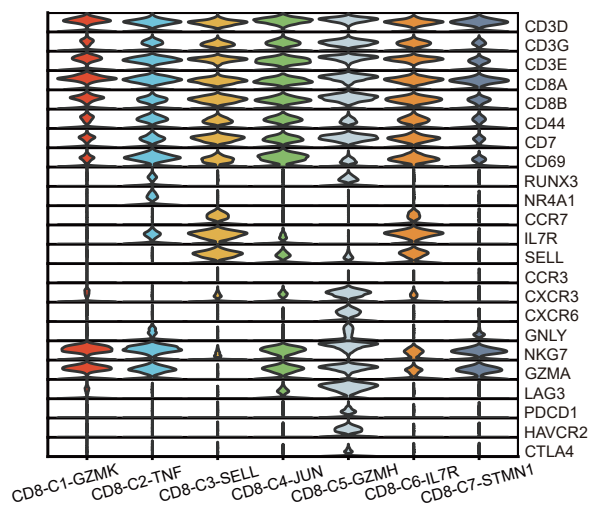

C

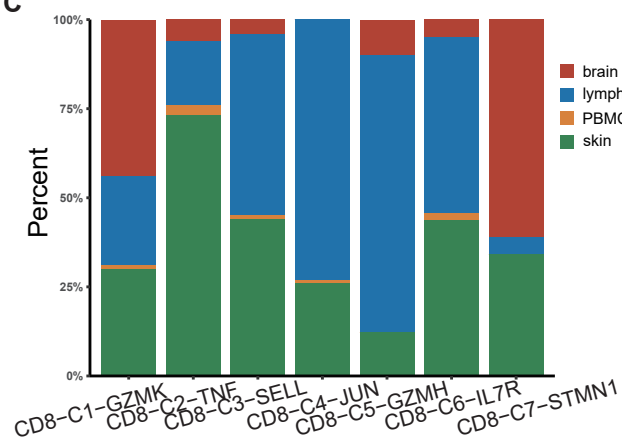

D

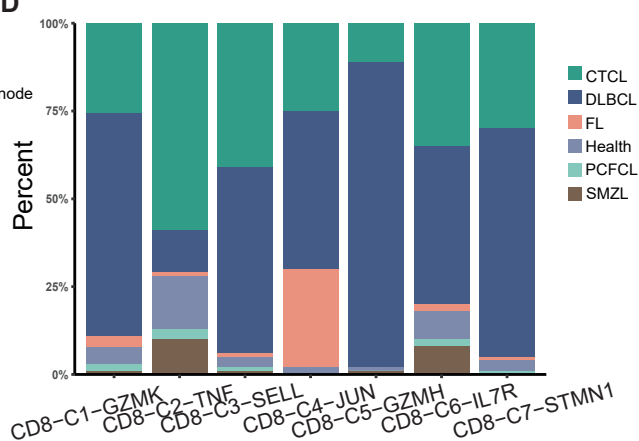

E

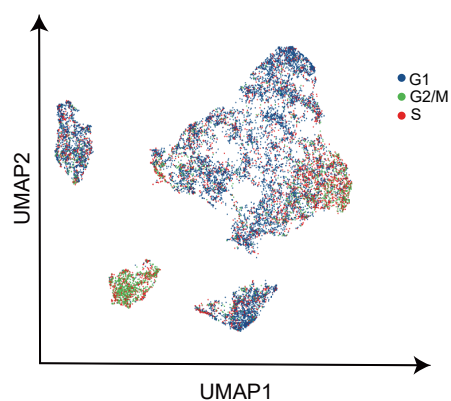

F

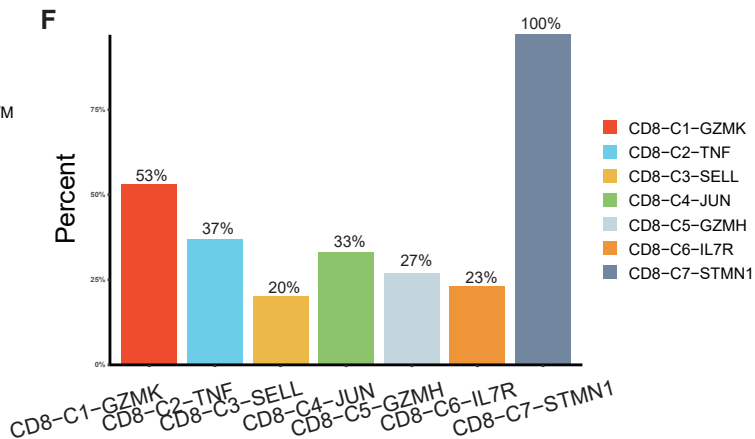

G

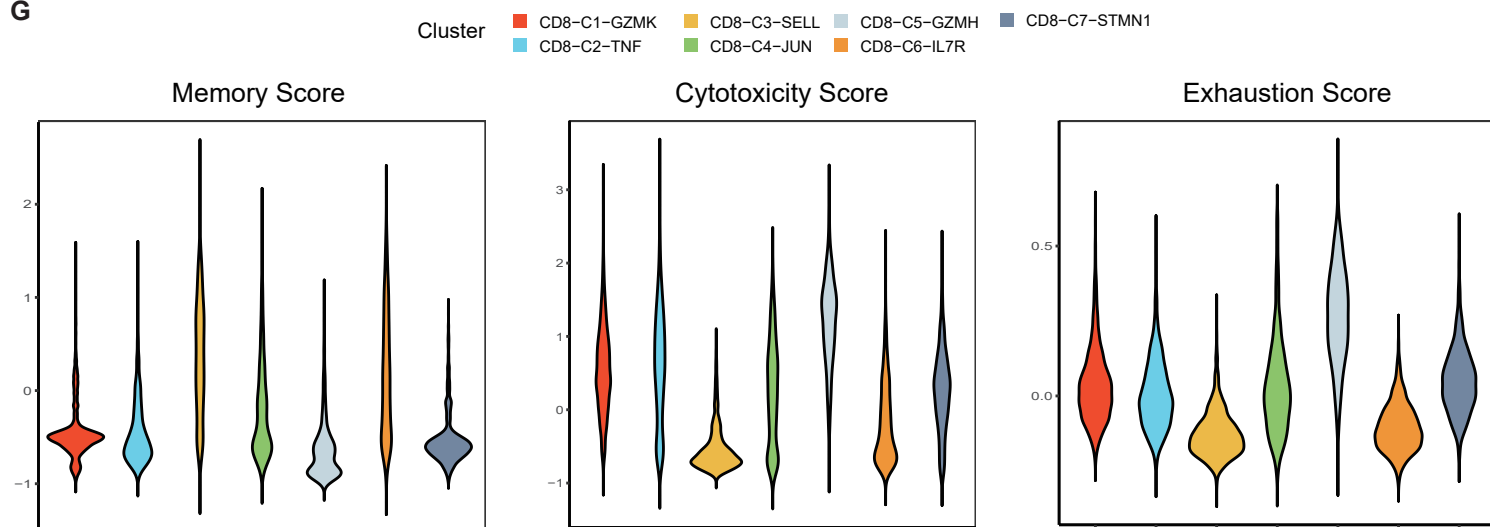

Supplement: Multimedia component 6 [file mmc6.pdf]

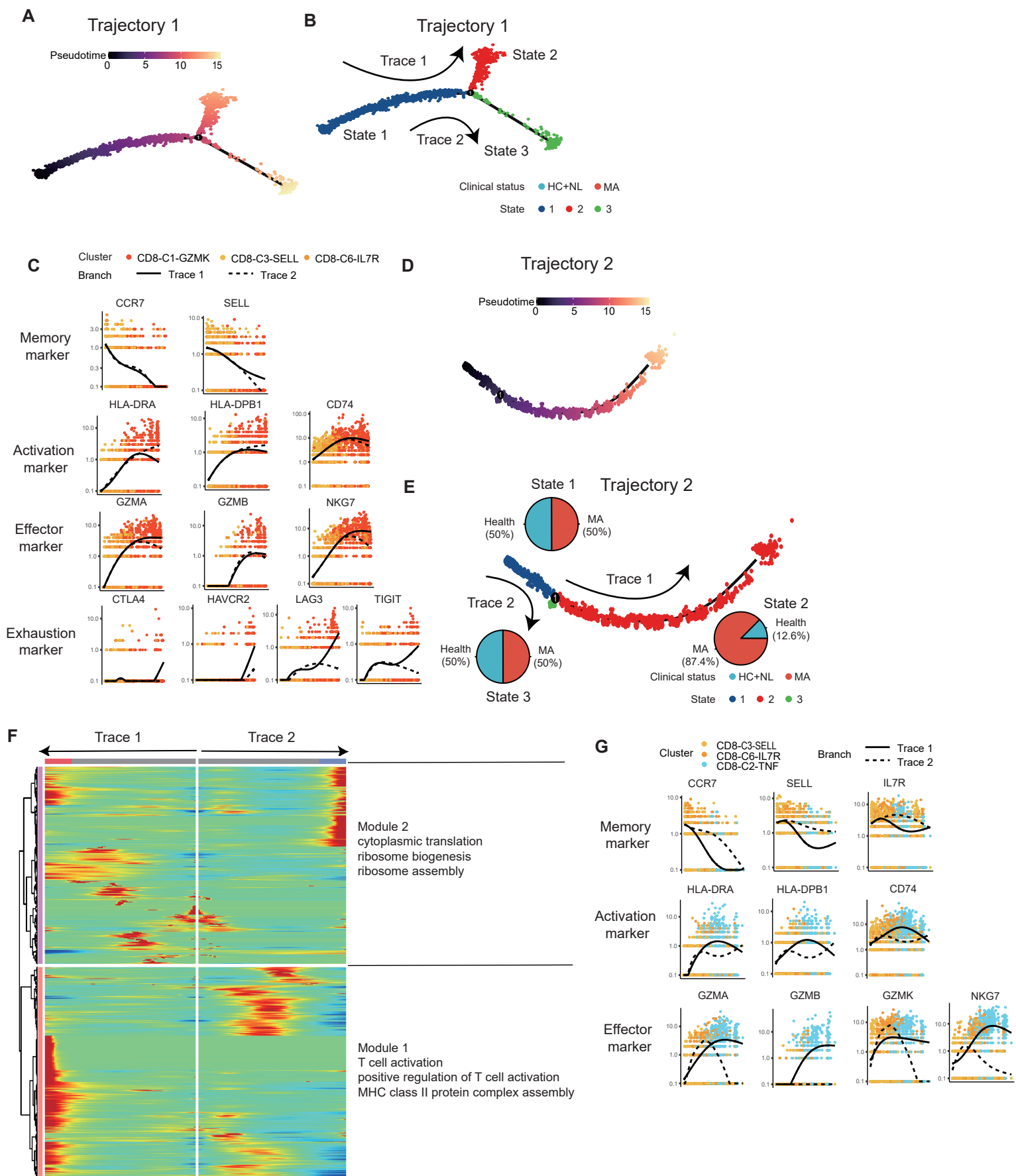

Supplement: Multimedia component 7 [file mmc7.pdf]

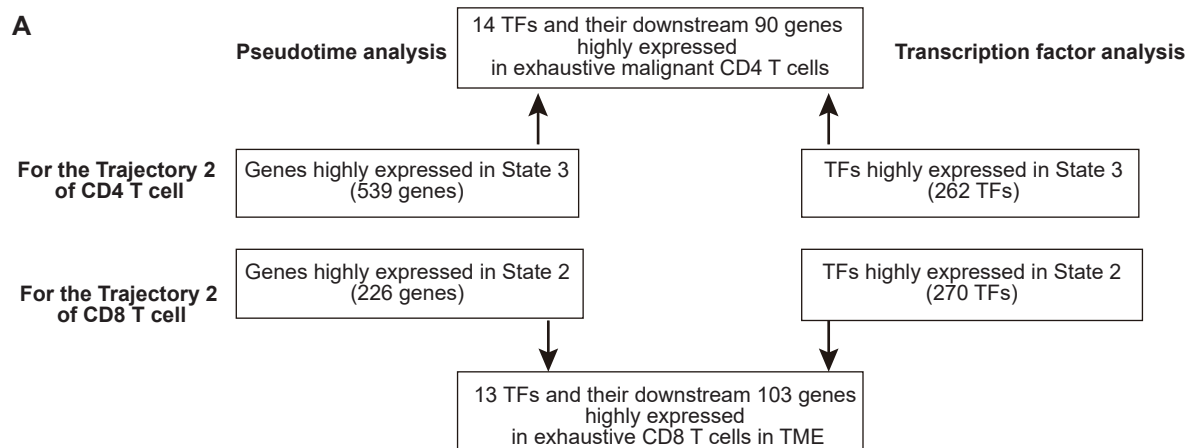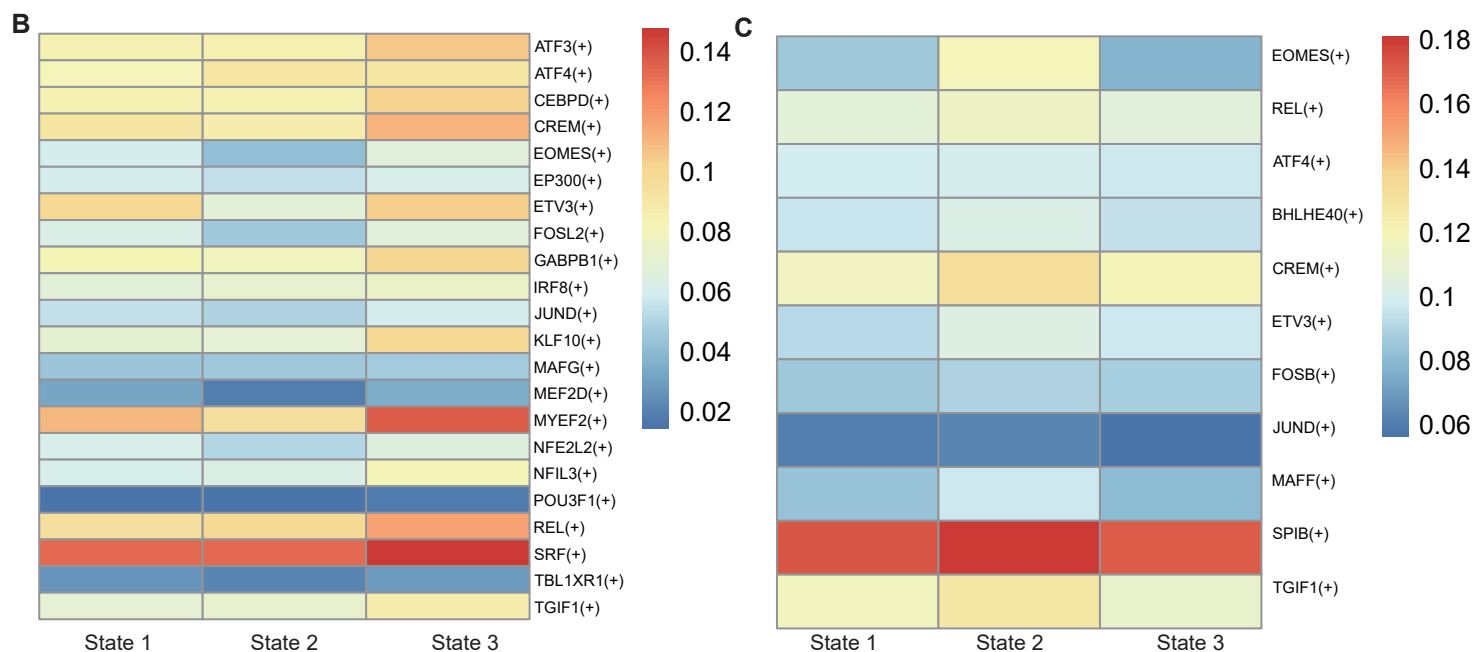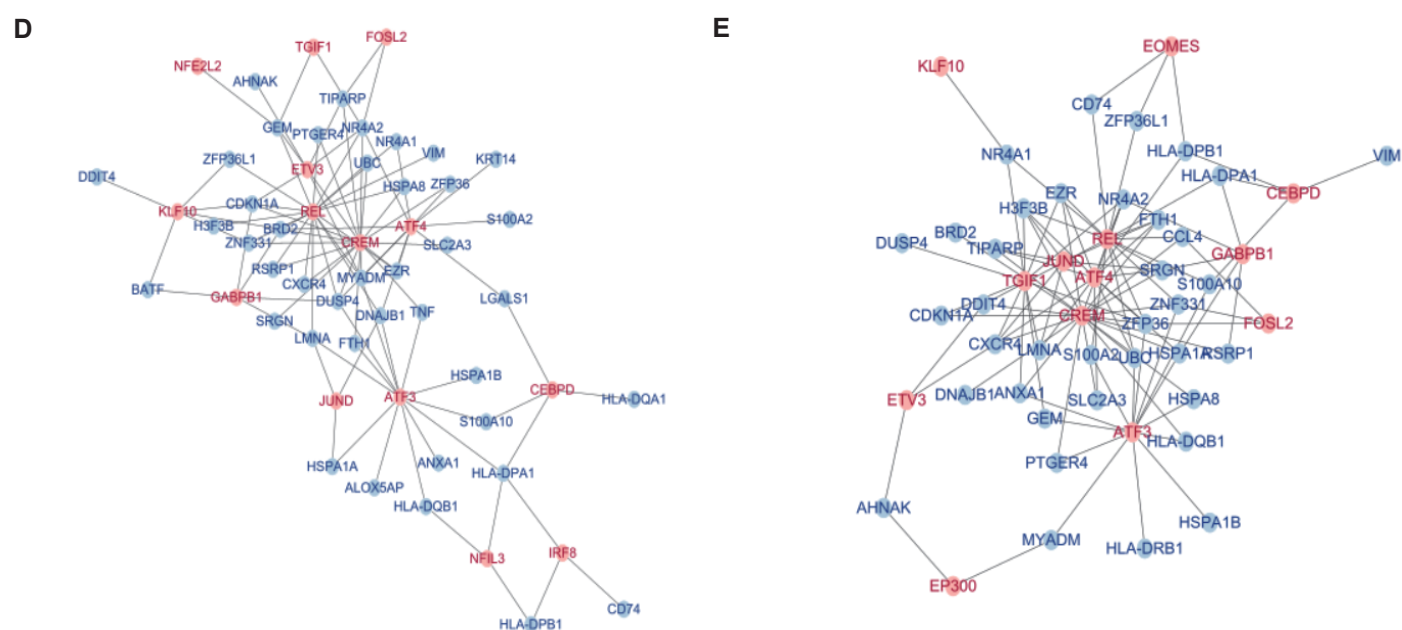

Supplement: Multimedia component 8 [file mmc8.pdf]
