# Supplementary material for: The single-cell landscape exploring abnormal T cell states and developmental trajectories in heterogeneous non-Hodgkin lymphoma
Source: Genes Dis. 2025 Aug 19;13(4):101812. doi: 10.1016/j.gendis.2025.101812 (PMC13015217; doi:10.1016/j.gendis.2025.101812)
Supplement: Multimedia component 9 [file mmc9.pdf]

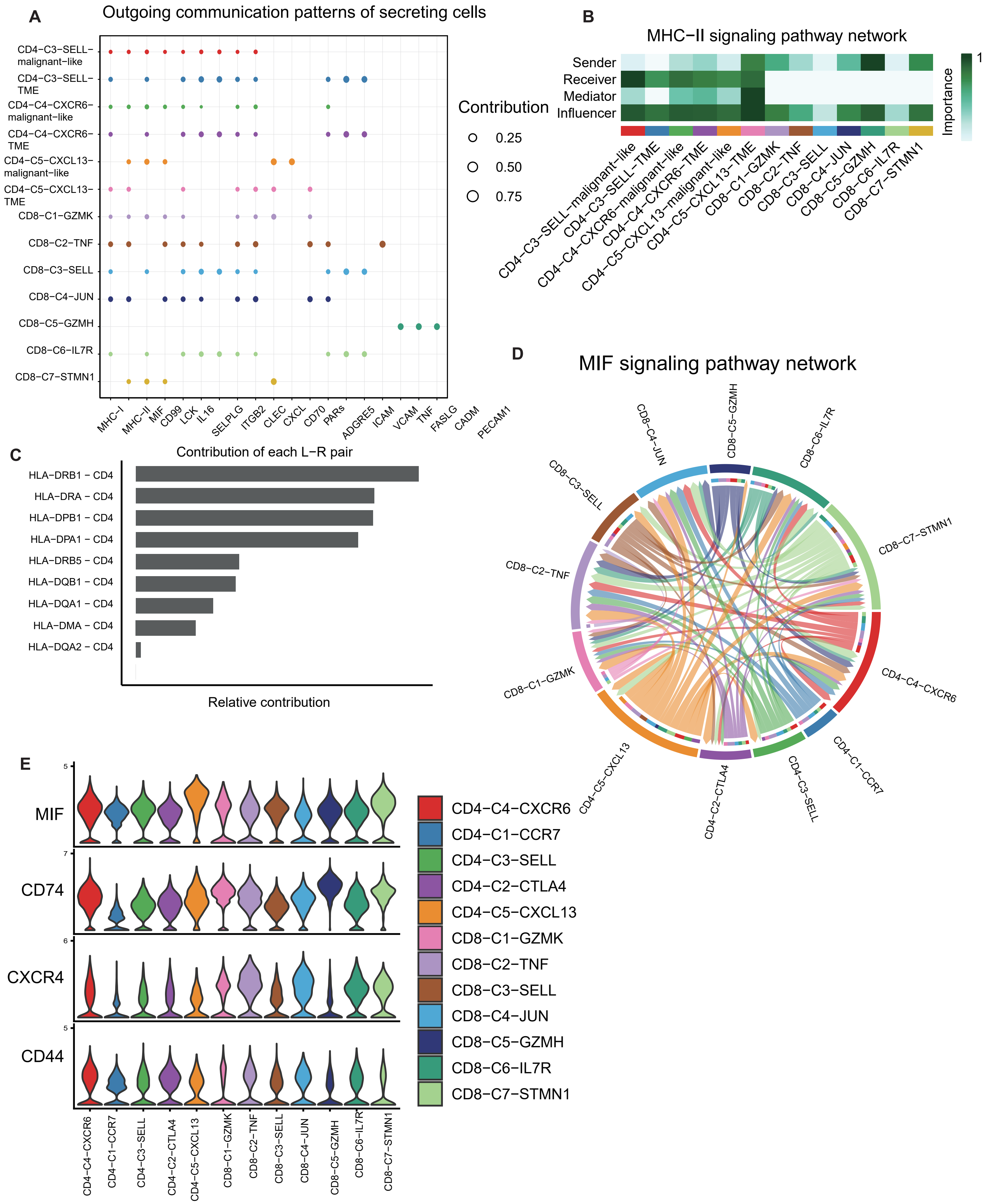
A
Outgoing communication patterns of secreting cells
CD4−C3−SELL−malignant−like
CD4−C3−SELL−TME
CD4−C4−CXCR6−malignant−like
CD4−C4−CXCR6−TME
CD4−C5−CXCL13−malignant−like
CD4−C5−CXCL13−TME
CD8−C1−GZMK
CD8−C2−TNF
CD8−C3−SELL
CD8−C4−JUN
CD8−C5−GZMH
CD8−C6−IL7R
CD8−C7−STMN1
MHC−I
MHC−II
MIF
CD99
LCK
IL16
SELPLG
ITGB2
CLEC
CXCL
CD70
PARs
ADGRE5
ICAM
VCAM
TNF
FASLG
CADM
PECAM1
Contribution
0.25
0.50
0.75
B
MHC−II signaling pathway network
Sender
Receiver
Mediator
Influencer
Importance
1
CD4−C3−SELL−malignant−like
CD4−C3−SELL−TME
CD4−C4−CXCR6−malignant−like
CD4−C4−CXCR6−TME
CD4−C5−CXCL13−malignant−like
CD4−C5−CXCL13−TME
CD8−C1−GZMK
CD8−C2−TNF
CD8−C3−SELL
CD8−C4−JUN
CD8−C5−GZMH
CD8−C6−IL7R
CD8−C7−STMN1
C
Contribution of each L−R pair
HLA−DRB1 − CD4
HLA−DRA − CD4
HLA−DPB1 − CD4
HLA−DPA1 − CD4
HLA−DRB5 − CD4
HLA−DQB1 − CD4
HLA−DQA1 − CD4
HLA−DMA − CD4
HLA−DQA2 − CD4
Relative contribution
D
MIF signaling pathway network
CD8−C5−GZMH
CD8−C4−JUN
CD8−C6−IL7R
CD8−C3−SELL
CD8−C7−STMN1
CD8−C2−TNF
CD4−C4−CXCR6
CD8−C1−GZMK
CD4−C1−CCR7
CD4−C5−CXCL13
CD4−C2−CTLA4
CD4−C3−SELL
E
MIF
CD74
CXCR4
CD44
5
7
6
5
CD4−C4−CXCR6
CD4−C1−CCR7
CD4−C3−SELL
CD4−C2−CTLA4
CD4−C5−CXCL13
CD8−C1−GZMK
CD8−C2−TNF
CD8−C3−SELL
CD8−C4−JUN
CD8−C5−GZMH
CD8−C6−IL7R
CD8−C7−STMN1
