# Supplementary material for: The single-cell landscape exploring abnormal T cell states and developmental trajectories in heterogeneous non-Hodgkin lymphoma
Source: Genes Dis. 2025 Aug 19;13(4):101812. doi: 10.1016/j.gendis.2025.101812 (PMC13015217; doi:10.1016/j.gendis.2025.101812)
Supplement: Multimedia component 10 [file mmc10.pdf]

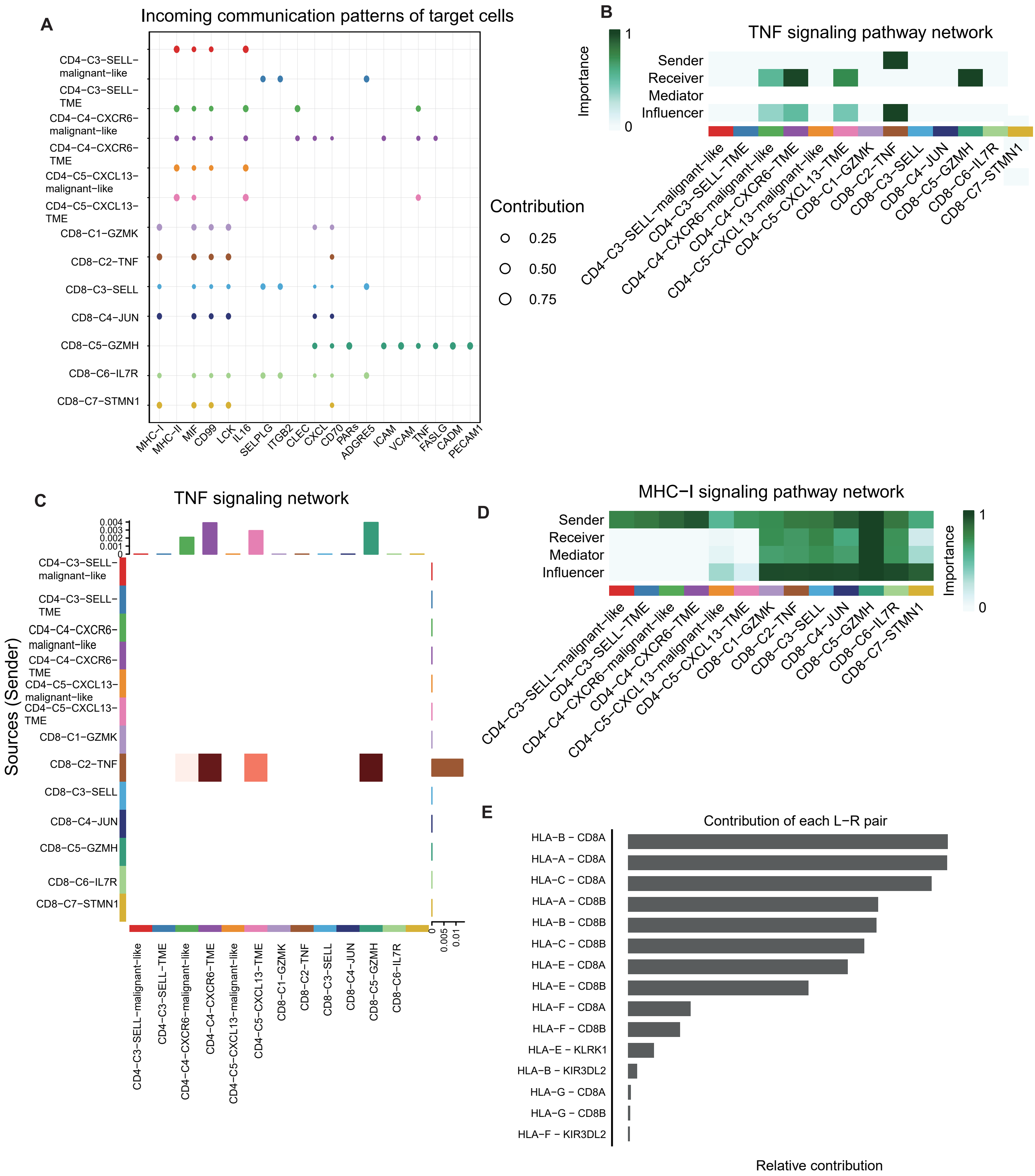
A
Incoming communication patterns of target cells
CD4−C3−SELL−malignant−like
CD4−C3−SELL−TME
CD4−C4−CXCR6−malignant−like
CD4−C4−CXCR6−TME
CD4−C5−CXCL13−malignant−like
CD4−C5−CXCL13−TME
CD8−C1−GZMK
CD8−C2−TNF
CD8−C3−SELL
CD8−C4−JUN
CD8−C5−GZMH
CD8−C6−IL7R
CD8−C7−STMN1
MHC−I
MHC−II
MIF
CD99
LCK
IL16
SELPLG
ITGB2
CLEC
CXCL
CD70
PARs
ADGRE5
ICAM
VCAM
TNF
FASLG
CADM
PECAM1
Contribution
0.25
0.50
0.75
B
TNF signaling pathway network
Importance
1
0
Sender
Receiver
Mediator
Influencer
C
TNF signaling network
Sources (Sender)
0.004
0.003
0.002
0.001
0
0.005
0.01
D
MHC−I signaling pathway network
E
Contribution of each L−R pair
HLA−B − CD8A
HLA−A − CD8A
HLA−C − CD8A
HLA−A − CD8B
HLA−B − CD8B
HLA−C − CD8B
HLA−E − CD8A
HLA−E − CD8B
HLA−F − CD8A
HLA−F − CD8B
HLA−E − KLRK1
HLA−B − KIR3DL2
HLA−G − CD8A
HLA−G − CD8B
HLA−F − KIR3DL2
Relative contribution
