# Supplementary material for: The single-cell landscape exploring abnormal T cell states and developmental trajectories in heterogeneous non-Hodgkin lymphoma
Source: Genes Dis. 2025 Aug 19;13(4):101812. doi: 10.1016/j.gendis.2025.101812 (PMC13015217; doi:10.1016/j.gendis.2025.101812)
Supplement: Multimedia component 18 [file mmc18.docx]

**The single-cell transcriptomic landscape of non-Hodgkin lymphoma**

scRNA-seq data were available from ten published cohorts (Table S1). These cohorts included six types of non-Hodgkin lymphoma, cutaneous T cell lymphoma (CTCL), primary central nervous system lymphoma (PCNSL), primary cutaneous follicle center cell lymphoma (PCFCL), follicular lymphoma (FL), diffuse large B cell lymphoma (DLBCL), and splenic marginal zone lymphoma (SMZL) (Figure S1B). These data covered four tissue specimens (skin, brain, peripheral blood mononuclear cell (PBMC), and lymph node) (Figure S1C). A total of 77.9% of the cells were from malignant tissues, whereas the remaining cells originated from healthy tissues (Figure S1D). We analyzed cells from these cohorts after reintegration (Figure S1A).

Unsupervised clustering analysis grouped NHL cells into eleven clusters (Figure S1E). The distinct expression patterns of the canonical markers allowed us to annotate these clusters, which were further confirmed using the cell type identification package-“SingleR”. T cells were clusters characterized by high expression of *CD3D*, *CD3G*, and *CD3E* (Figure S1F). Three clusters of B cells were identified by *CD79A* and *CD79B*. Other types of immune cells (NK cells and DC cells) and stromal cells (chondrocytes, endothelial cells, epithelial cells & keratinocytes, and stromal mesenchymal cells (MSCs)) were identified.
