# Supplementary material for: The single-cell landscape exploring abnormal T cell states and developmental trajectories in heterogeneous non-Hodgkin lymphoma
Source: Genes Dis. 2025 Aug 19;13(4):101812. doi: 10.1016/j.gendis.2025.101812 (PMC13015217; doi:10.1016/j.gendis.2025.101812)
Supplement: Multimedia component 19 [file mmc19.docx]

**CD4^+^ T cells exhibit origin-specific transcriptomic features and functions.**

To comprehensively categorize the populations of T cells and their crosstalk, we selected CD4^+^ T cells and CD8^+^ T cells from the T cell cluster shown in Supplementary Figure 1 based on the expression of CD4^+^ and CD8^+^. We generated two additional transcriptomic maps of CD4^+^ T cells and CD8^+^ T cells from non-Hodgkin lymphoma (NHL) cells (Figure A and Figure S4A).

CD4^+^ T cells were grouped into five subtypes (Figure A) according to distinct transcriptomic patterns (Figure S2A) and annotated by the expression of canonical T cell markers (Figure S2B). CD4-C1-CCR7 and CD4-C3-SELL were two dominant clusters characterized by the high expression of memory-associated markers, *CCR7*, *IL7R*, and *SELL* (Figure S2B). Memory T cells specifically express *CCR7*, *TCF7*, *LEF1*, *SELL* and *IL7R*; thus, memory scores were generated based on these four genes. Their memory features were confirmed by the highest memory scores (Figure S2E). The tissue heterogeneity was detected (Figure S2C). CD4-C1-CCR7 cells obtained from mainly peripheral blood mononuclear cells (PBMCs), while most of CD4-C4-CXCR6 and CD4-C5-CXCL13 cells originated from the skin. CD4-C2-CTLA4 and CD4-C3-SELL tended to be B-cell specific, whereas cells of CD4-C4-CXCR6 and CD4-C5-CXCL13 were mainly from T-cell NHL (Figure S2D). Moreover, cells from healthy tissues were the most in CD4-C1-CCR7. CD4-C2-CTLA4 highly expressed Treg markers (*FOXP3*, *CTLA4*, and *LAG3*). According to our clustering at the single-cell level, CD4-C1-CCR7 and CD4-C2-CTLA4 were viewed as CD4^+^ T cells in the tumor microenvironment (TME). CD4-C3-SELL, CD4-C4-CXCR6, and CD4-C5-CXCL13 were described as malignant-like CD4^+^ T cells. To evaluate the proliferative potential of these clusters, cell cycle scores, which were previously shown to denote the G1, G2/M, and S phases, were used to infer the cell cycle phases. G1 comprises the main types of CD4-C1-CCR7, CD4-C2-CTLA4, CD4-C3-SELL, and CD4-C4-CXCR6 (Figure S2F). Meanwhile, all the CD4-C5-CXCL13 cells were cycling (Figure S2G). The higher proliferation of CD4-C5-CXCL13 means higher malignancy compared to CD4-C5-CXCR6.

In consideration to further identify functional CD4^+^ T cell subtypes, differential expression analysis was done among B-cell and T-cell NHL cells and healthy cells. CD4^+^ T cells from different origins exhibited distinguished transcriptomic features (Figure C). B-cell NHL-specific CD4^+^ T cells highly expressed the important inhibitory receptor, programmed death-1 (PD-1) (encoded by *PDCD1*), and co-inhibitory receptor *LAG3*. These findings illustrated that B-cell NHL-specific CD4^+^ T cells may play an important role in restricting anti-tumor CD8^+^ T cells in the TME. Differently, genes encoding mitochondrial ATP synthases (*ATP4MG*, *ATP5MC2*, *ATP5MC3*, *ATP5F1B*, *ATP5F1C*, and *ATP5MF*) were highly expressed in CD4^+^ T cells from T-cell NHLs. These results indicate that T-cell NHLs tend to increase oxidative phosphorylation. The top 100 differentially expressed genes formed three specific gene sets, featuring malignancy, TME and health (Table S2-4). As expected, CD4-C5-CXCL13 and CD4-C5-CXCR6 had the highest malignancy scores and CD4-C1-CCR7 had the highest health score (Figure B). The remaining clusters showed the transition state.
