# Supplementary material for: The single-cell landscape exploring abnormal T cell states and developmental trajectories in heterogeneous non-Hodgkin lymphoma
Source: Genes Dis. 2025 Aug 19;13(4):101812. doi: 10.1016/j.gendis.2025.101812 (PMC13015217; doi:10.1016/j.gendis.2025.101812)
Supplement: Multimedia component 20 [file mmc20.docx]

**CD4^+^ T cells from non-Hodgkin lymphoma exhibited malignancy-specific developmental trajectories.**

To further investigate the dynamic transitional process of CD4^+^ T cells of non-Hodgkin lymphoma and compare developmental differences of cells from tumor microenvironment (TME) and malignant tissues, we first applied Monocle 3 to trace the fates of all CD4^+^ T cells and reconstruct cell lineages (Figure S3A). Trajectory 1 was determined to initiate with CD4-C1-CCR7 as the beginning, and reached a state of CD4-C2-CTLA4 (Figure S3B-C). Trajectory 2 was determined to start from CD4-C3-SELL, and finally reached a terminally differentiated state of CD4-C4-CXCR6 and CD4-C5-CXCL13 (Figure D and Figure S3E).

For Trajectory 1, the expression of memory marker genes (*CCR7*, *SELL*, and *IL7R*) and T-cell inhibition genes (*CTLA4*, *FOXP3*, *HAVCR2*, *LAG3*, and *PDCD1*) across the trajectory confirmed the capture of the transition from memory-like cells to Treg-like cells (Figure S3E). In total, 3,871 genes were identified to change significantly through the pseudotime (p-value < 0.01, fold change > 2) and were grouped into three modules of co-expressed genes that showed similar patterns of expression across differentiation (Figure S3F). Gene ontology (GO) term enrichment for biological processes associated with each gene module revealed the relative order of biological events during memory to Treg development. Processes upregulated at the start of the trajectory included ribonucleoprotein complex biogenesis, ribosome biogenesis, ribosome assembly (module 3), and these were then followed by mitotic sister chromatid segregation, mitotic nuclear division, and DNA replication (module 1), indicating the progression of cell proliferation. Meanwhile, genes associated with histone modification, negative regulation of leukocyte proliferation, and negative regulation of T cell proliferation (module 2) increasingly expressed following the trajectory. According to the results from the evaluation of the health score and TME score, state 2 and state 3 showed similar status as TME cells (Figure S3D). Thus, the trail from State 1 to State 2 or State 3 described the inhibitory immune environment of anti-tumor T-cell activation in non-Hodgkin lymphoma (NHL) TME, which likely contributes to the relapse.

Trajectory 2 was determined and the cells from CD4-C3-SELL, CD4-C4-CXCR6, and CD4-C5-CXCL13 were matched to three developmental states with one potential differential branched point by sorting the significant differentially expressed genes across these three clusters (Figure D). Whether State 2 and State 3 had higher malignancy scores and lower health scores (Figure E and Figure S3H). It illustrated that Trace 1 and Trace 2 of Trajectory 2 both exhibited malignant traces from memory-like cells to highly malignant cells.

Along Trajectory 2 of CD4^+^ T cells, the expression of memory marker genes (*CCR7*, *SELL*, and *IL7R*) decreased (Figure S3I). CD4-C3-SELL developed to significantly proliferate, finally differentiated to be CD4-C5-CXCL13 with the characteristics of the highest proportion of cycling cells (Figure S2G). Similarly, genes identified as Module 1, including mitotic nuclear division, DNA replication, and nuclear division, are highly expressed at the end (Figure S3J). Moreover, the malignancy score of State 2 was higher than that of State 3 (Figure E). These results captured the abnormal activation of memory-like cells to malignant cells, along with the upregulation of activation markers (*HLA-DRA*, *HLA-DPB1*, *CD74*, and *IL2RA*) (Figure S3I). Simultaneously, exhaustion markers (*CTLA4*, *LAG3*, *TIGIT1*, and *FOXP3*) downregulated in State 2 while State 3 had the opposite effect. The decreased expression of exhaustion genes illustrated that cells from State 2 had persistent proliferative potential with high malignancy. These cells probably contribute to relapsed problems.
