# Supplementary material for: The single-cell landscape exploring abnormal T cell states and developmental trajectories in heterogeneous non-Hodgkin lymphoma
Source: Genes Dis. 2025 Aug 19;13(4):101812. doi: 10.1016/j.gendis.2025.101812 (PMC13015217; doi:10.1016/j.gendis.2025.101812)
Supplement: Multimedia component 21 [file mmc21.docx]

**CD8^+^ T cells in the tumor microenvironment of non-Hodgkin lymphoma showed efficient cytotoxicity with high exhaustive potential.**

To reveal the potential functional subtypes of CD8^+^ T cells, we performed dimensionality reduction and clustering of T cells expressed *CD8A* and *CD8B*. Seven CD8^+^ T cells in tumor microenvironment (TME) were identified (Figure S4A). Cells from CD8-C1-GZMK and CD8-C7-STMN1 were mainly from brains, whereas cells from lymph nodes occupied CD8-C4-JUN and CD8-C5-GZMH (Figure S4C). In particular, cells from skin tissues were largely predominant in CD8-C2-TNF. CD8-C3-SELL and CD8-C5-IL7R were two dominant clusters characterized by the high expression of *SELL*, *CCR7*, *IL7R*, *TGF7*, and *LEF1* (Figure S4B). These two clusters carried low levels of cytokines and effector genes. Markedly high expression of cytotoxic activity related genes (*NKG7*, *PRF1*, and *GZMK*) was observed in CD8-C1-GZMK, CD8-C2-TNF, CD8-C4-JUN, CD8-C5-GZMH, and CD8-C7-STMN1.

To explore the proliferative potential of CD8^+^ T cells, cell cycle scores were evaluated and the results showed that a significant fraction of cells from CD8-C1-GZMK and CD8-C7-STMN1 were observed to be cycling. However, CD8-C2-TNF, CD8-C3-SELL, CD8-C4-JUN, CD8-C5-GZMH, and CD8-C6-IL7R largely remained in the G1 state (Figure S4E). Pivotal markers of memory, cytotoxicity, and exhaustion were selected to evaluate cellular function and status. Marker genes were listed in detail in Table S5. Particularly, CD8-C5-GZMH had the highest cytotoxicity score and the highest exhaustion score (Figure S4G).

To reveal the developmental stages of the CD8^+^ T cells, the pseudotime analysis was achieved by Monocle 3. The results displayed two developmental trajectories, one was from CD8-C3-SELL through CD8-C6-IL7R-CD3 to CD8-C1-GZMK, and another destination was CD8-C2-TNF (Figure F). For Trajectory 1, three states were identified (Figure G, Figure S5A and Figure S5B). In State 2, genes enriched as Module 1 (T cell activation, T cell proliferation, and T cell differentiation) and Module 2 (T cell receptor signaling pathway, T cell activation, and T cell proliferation) are highly expressed, illustrating that memory CD8^+^ T cells in TME attend to be activated by tumor burdens (Figure H). The expression of activation markers (*HLA-DRA*, *HLA-DPB1*, and *CD74*) and effector markers (*GZMA*, *GZMB*, and *NKG7*) increased with the reduction of exhaustion markers (*CTLA4*, *HAVCR2*, *LAG3*, and *TIGIT*) (Figure S5C).

Similarly, the developmental trajectory of CD8-C2-TNF, CD8-C3-SELL, and CD8-C6-IL7R was reanalyzed using Monocle 2. When tumor burdens increased, the cytotoxicity of CD8^+^ T cells enhanced along Trace 1 (Figure I) of Trajectory 2, which was confirmed by upregulation of genes in Module 1 (T cell activation, positive regulation of T cell activation, MHC class II protein complex assembly) (Figure S5F). However, branched expression analysis modeling showed the differences of markers along Trace 1 and Trace 2. Compared to Trace 1, effector markers (*GZMA*, *GZMB*, *GZMK*, and *NKG7*) ultimately decreased (Figure S5G). And the exhaustive signals increased (Figure J).
