# Supplementary material for: The single-cell landscape exploring abnormal T cell states and developmental trajectories in heterogeneous non-Hodgkin lymphoma
Source: Genes Dis. 2025 Aug 19;13(4):101812. doi: 10.1016/j.gendis.2025.101812 (PMC13015217; doi:10.1016/j.gendis.2025.101812)
Supplement: Multimedia component 22 [file mmc22.docx]

**Transcriptomic networks were identified to cause T cell exhaustion in non-Hodgkin lymphoma.**

To further explore the regulation network to promote exhaustion of both malignant CD4^+^ T cells and CD8^+^ T cells in the tumor microenvironment (TME), we performed the transcriptomic factor (TF) enrichment analysis. We selected key genes promoting exhaustion calculated through pseudotime analysis and TFs identified as regulons of these genes (Figure S6A). For Trajectory 2 of CD4^+^ T cells, State 3 exhibited more exhaustive characteristics than State 2 (Figure S3I). Exhaustion markers showed higher expression in State 2 than in State 3 along Trajectory 2 of CD8^+^ T cells (Figure J). Then we compared State 2 and State 3 in two trajectories, respectively, to find the important TFs in the transition to exhaustion. Fourteen TFs in CD4^+^ T cells and thirteen TFs in CD8^+^ T cells were finally selected (Figure 6B-C). Our results showed that ATF4, CREM, EOMES, ETV3, JUND, and REL play significant roles in the induction of T cell exhaustion (Figure S6D-E). These TFs and genes were found to be possible novel targets to suppress the exhaustion of T cells in TME and promote the exhaustion of T-cell lymphoma, finally developing anti-tumor efficacy and reducing relapsed or refractory problems.
