# Supplementary material for: The single-cell landscape exploring abnormal T cell states and developmental trajectories in heterogeneous non-Hodgkin lymphoma
Source: Genes Dis. 2025 Aug 19;13(4):101812. doi: 10.1016/j.gendis.2025.101812 (PMC13015217; doi:10.1016/j.gendis.2025.101812)
Supplement: Multimedia component 23 [file mmc23.docx]

**MHC-II molecules in CD8^+^ T cells in the tumor microenvironment may cause the escape of malignant T cells.**

To decipher the ligand-receptor interactions in CD4^+^ T cells in tumor microenvironment (TME) and tumors with CD8^+^ T cells in TME, we performed cell-cell interaction analyses. The results from CellChat indicated that CD4^+^ T cells in TME and tumors had various immune interactions with CD8^+^ T cells. We observed that although cells from TME and tumors were identified as the same subclusters, their outgoing communication patterns differed (Figure S7A and Figure S8A). MHC-II signaling pathway significantly contributed to the communication between malignant-like CD4^+^ T cells and CD8^+^ T cells than CD4^+^ T cells in TME (Figure S7B). Malignant-like CD4^+^ T cells were more important as receivers for MHC-II signaling pathway network than CD4^+^ T cells in TME. And CD8-C5-GZMH was the vital sender, which simultaneously had the highest cytotoxicity score and exhaustion score (Figure S4G).

The interactions based on the MHC-II signaling pathway between CD4^+^ T cells and CD8^+^ T cells were mediated mainly by CD8-C5-GZMH, CD8-C4-JUN, and CD8-C7-STMN1. Notably, among all known ligand-receptor pairs, MHC-II signaling is dominated by HLA-DRB1 ligand and CD4 receptor (Figure S7C). Previous results showed that CD8^+^ T cells mainly attacked malignant-like CD4^+^ T cells through MHC-II signaling pathway. However, the cell-cell communications were not dominated by the typical tumor suppressor signaling, TGFb signaling pathway (not shown). Instead, TNF signaling was shown to be important in the communications between CD8-C5-GZMH and CD4^+^ T cells (Figure S8B-C). Simultaneously, the vital killer CD8-C5-GZMH expressed high exhaustive signals, which means that they were poor in persistence. The malignant-like CD4^+^ T cells possibly escape the cytotoxicity of CD8^+^ T cells to cause refractory problems. CD4^+^ T cells in TME of B-cell lymphoma also upregulated the ligand HLA-B to attach to *CD8A* to stimulate MHC-I signaling pathway to speed the exhaustion of cytotoxic CD8^+^ T cells (Figure S8D-E). Besides, CD4-C5-CXCL13, which has the highest malignancy score and the most cycling cells, highly expressed macrophage migration inhibitory factor (MIF) to communicate with anti-tumor CD8^+^ T cells mainly through MIF-CD74 (Figure K and Figure S7E), probably causing the abnormal activation of anti-tumor CD8^+^ T cells and then leading to poor persistence.
